# Factors influencing inconsistent leg length discrepancy in dysplastic hip osteoarthritis: a retrospective study

**DOI:** 10.1186/s12891-022-05348-z

**Published:** 2022-04-23

**Authors:** Genta Takemoto, Yusuke Osawa, Taisuke Seki, Yasuhiko Takegami, Satoshi Ochiai, Daisaku Kato, Shiro Imagama

**Affiliations:** Department of Orthopedic Surgery, Nagoya University Graduate School of Medicine, 65 Tsurumai-cho, Showa-ku, Nagoya City, 466-8550 Japan

**Keywords:** Leg length discrepancy, Dysplastic hip osteoarthritis, Perceived, Pelvic oblique angle, Subluxation

## Abstract

**Background:**

We aimed to examine the inconsistency between radiographic leg length discrepancy (R-LLD) and perceived LLD (P-LLD) in patients with dysplastic hip osteoarthritis and to evaluate the factors that can cause such inconsistency.

**Methods:**

We conducted a retrospective study on 120 patients. An inconsistent LLD was defined as a condition in which the P-LLD was shorter than the R-LLD by > 5 mm. We compared relevant data on the general characteristics of the patients and the radiological findings between consistent (group E, 92 cases [76.7%]) and inconsistent LLDs (group S, 28 cases [23.3%]).

**Results:**

The number of patients with a history of hip surgery on the affected side and the Japanese Orthopedic Association classification pain scores were significantly higher in group S than in group E (32.1% vs. 10.8%, respectively; *P* = 0.015, and 21.7 ± 7.0 vs. 17.5 ± 8.2, respectively; *P* = 0.036). The pelvic oblique angle and length of the R-LLD were significantly higher in group S than in group E (2.9 ± 2.5° vs. 0.3 ± 2.3°, respectively; *P* < 0.01, and 17.2 ± 8.9 mm vs. 6.3 ± 8.4 mm, respectively; *P* < 0.01). Multivariate logistic analysis revealed that the pelvic oblique angle (odds ratio [OR]: 1.80, 95% confidence interval [CI]: 1.28–2.52; *P* < 0.01) and length of the R-LLD (OR: 2.75, 95% CI: 1.24–6.12; *P* = 0.013) were independent risk factors of inconsistent LLD.

**Conclusion:**

The pelvic oblique angle and a long R-LLD were independent risk factors of inconsistent LLD in patients with dysplastic hip osteoarthritis. Therefore, hip surgeons should consider P-LLD rather than R-LLD to understand the need for conservative intervention.

## Background

Dysplastic hip osteoarthritis (DHO) causes joint pain, limits range of motion (ROM) and activities of daily living, and impairs the quality of life (QOL) [1]. With the progression of DHO, the femoral head moves superolaterally and causes leg length discrepancy (LLD). LLD, derived from the hip joint, also impairs the patients’ QOL [2, 3]. There are many cases of radiographic LLD (R-LLD) in severe DHO [2].

It has also been confirmed that adjacent joints, such as the knee joint and lumbar spine, are damaged by LLD [4–6]. Therefore, it is common to adjust the LLD by using a shoe lift. Any discussion of LLD should include both R-LLD and perceived LLD (P-LLD) [7, 8], but there are no reports on whether it is necessary to use a shoe lift in consideration of either the R-LLD or P-LLD. In fact, in clinical practice, cases in which P-LLD was not observed despite the presence of R-LLD are commonly encountered [9]. However, there is no consensus on the reasons behind the inconsistency between the R-LLD and P-LLD.

In this study, the following clinical questions were addressed: (1) how often do inconsistent R-LLD-and-P-LLD occur in patients with DHO? and (2) what are the risk factors for an inconsistency between the P-LLD and R-LLD?

## Methods

### Patients

This study was based on a retrospective chart review and was approved by our institution’s Review Board. All patients provided their written informed consent to participate. We examined 176 patients who underwent THA for DHO at our hospital between April 2018 and March 2020. Patients were excluded if they had (1) a history of surgery in the lumbar region, knee, and lower legs (*n* = 21); (2) an apparent lower limb malalignment (*n* = 3); (3) severe knee osteoarthritis (*n* = 11); (4) severe acetabular dysplasia, such as Crowe type 3 or 4 (*n* = 10); (5) P-LLD that is longer than the R-LLD by ≥ 5 mm (*n* = 8); and (6) dementia or a hearing defect (*n* = 3). Based on these criteria, 120 cases were included in this study.

### Definition and grouping of inconsistent R-LLD and P-LLD

**Fig. 1:**
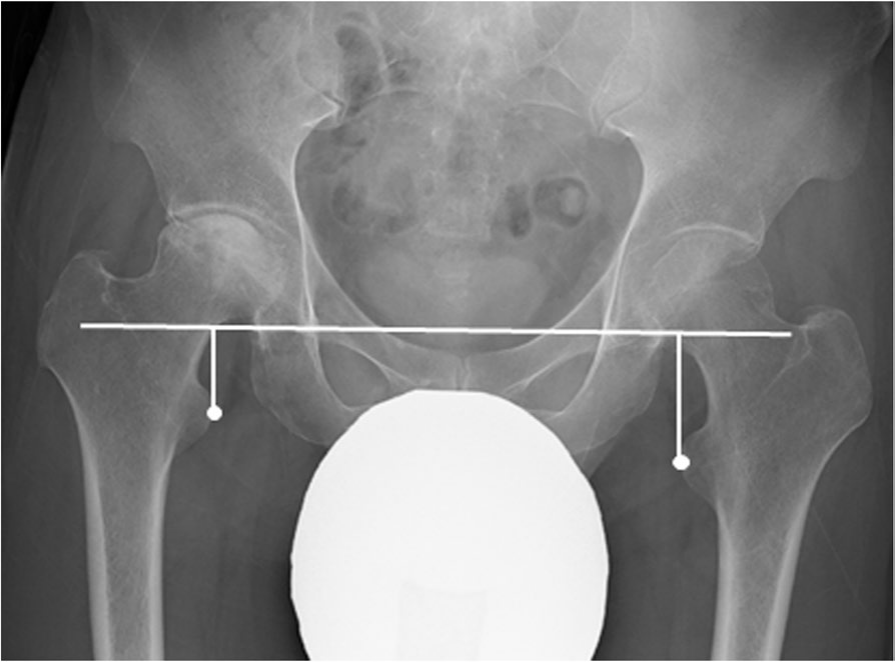
Preoperative anteroposterior pelvis radiograph measurement. Measured preoperative radiographic leg length discrepancy is 12 mm

The measurement of R-LLD was performed according to the method described by Woolson et al. [10]; a pelvic reference line was drawn through the most inferior part of the bilateral acetabular tears. Two lines parallel to the tear drops were drawn through the centers of the lesser trochanters. The difference in the perpendicular distance between the non-operative and operative sides was defined as the R-LLD (a positive value indicated that the hip joint on the operated side was shortened) (Fig. 1). This measurement, recorded in 1-mm units, was performed on the preoperative anteroposterior radiograph with the patient in a supine position, and the R-LLD was measured 4 weeks before surgery.

**Fig. 2:**
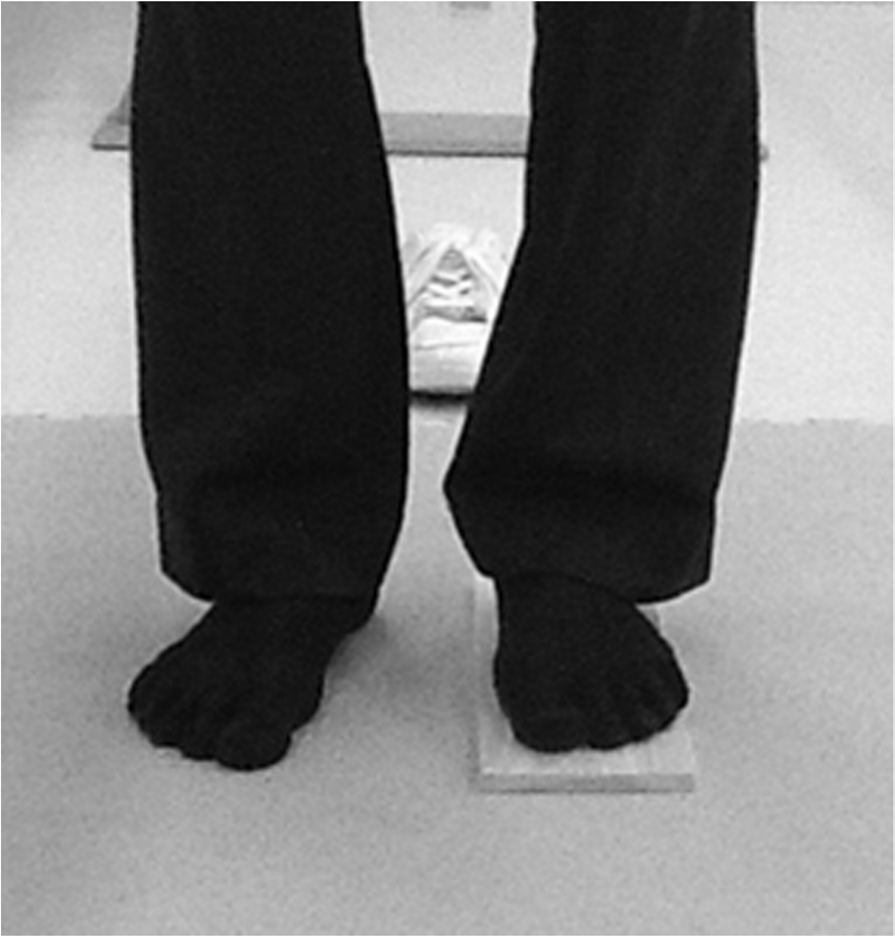
Measurement of “perceived LLD.” Perceived LLD was defined as the thickness of the block at the point at which the patient did not perceive LLD

Quantitative measurement of the P-LLD was performed according to the method described by Koga et al. [7]; the patient stepped barefoot onto a wooden spacer calibrated at 5-mm intervals (Fig. 2) to observe the height at which the patient perceived equal leg length, and the value was recorded in 5-mm units. During the step test examination, patients were instructed to stand without holding the handrail, with their back straight, and both the knees extended. A positive value indicated that the leg length on the operated side was short. The P-LLD was measured 4 weeks before surgery.

We subtracted the P-LLD from R-LLD, and assigned patients to one of two groups (groups E and S) based on the value obtained. Group E included patients with ≤ 5-mm difference between R-LLD and P-LLD or had the same R-LLD and P-LLD. Group S included patients whose P-LLD was shorter than R-LLD by > 5 mm. Eight patients with P-LLD longer than R-LLD by ≥ 5 mm were excluded from this study.

### Clinical evaluation

Background data including age, sex, body mass index (BMI), contralateral hip condition, history of surgery of the affected hip, and history of contralateral hip surgery were extracted from the patients’ medical records.

Hip joints were clinically evaluated using the scoring system proposed by the Japanese Orthopedic Association (JOA) [11] and the Japanese Orthopedic Association Hip-Disease Evaluation Questionnaire (JHEQ) [12]. The JOA hip score has been widely used in Japan to evaluate hip diseases. It consists of four categories: pain (0–40 points), ROM (0–20 points), gait ability (0–20 points), and the ability to perform activities of daily living (0–20 points). The 21-item JHEQ is a patient-reported outcomes tool, which evaluates pain, movement, and mental status; each subscale is scored on a 0–28-point range, with higher scores indicating a better QOL.

### Radiographic evaluation

**Fig. 3:**
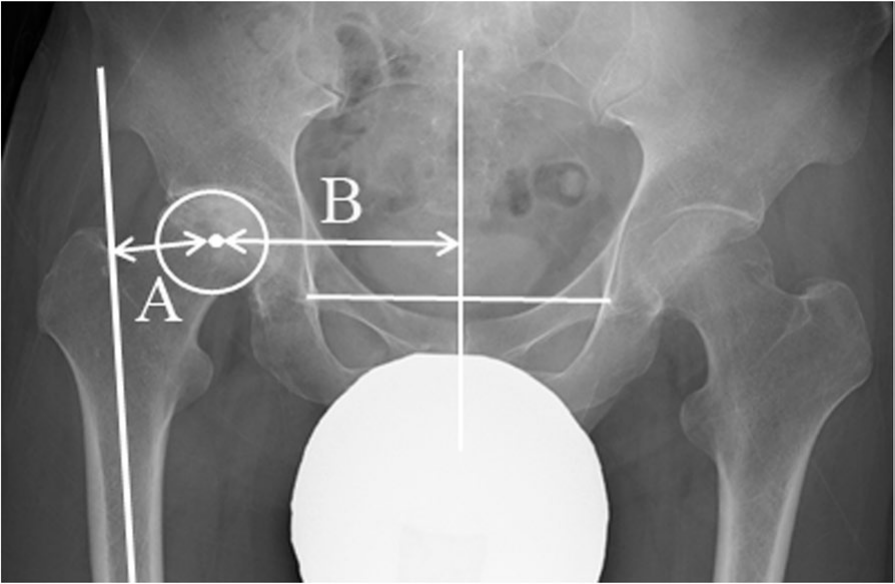
Measurement of grovel femoral offset (GFO). The preoperative GFO was measured by adding the distance between the longitudinal axis of the femur and the centre of the femoral head (A) to the distance between the centre of the femoral head and a perpendicular line passing through the pubic symphysis (B). GFO was defined as A + B

**Fig. 4:**
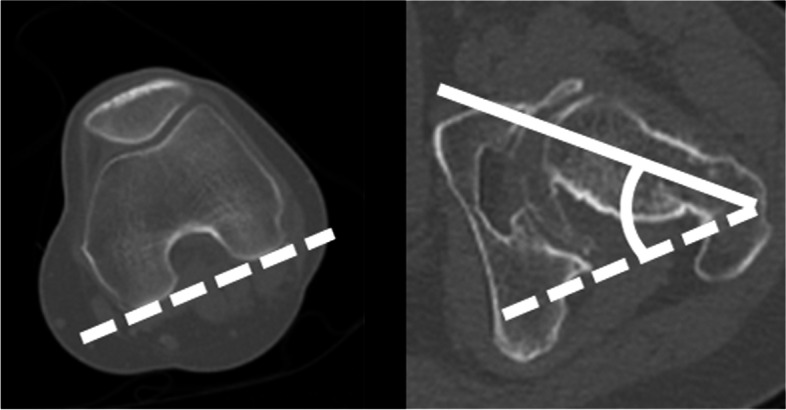
Measurement of anatomical femoral anteversion. Anatomical femoral anteversion was defined as the angle between the femoral neck axis (solid white line) and the posterior condylar plane (dotted white line)

The radiographis parameters used were chosen based on the reports by Koga et al. [7], Fujita et al. [9], and Kabata et al. [13]. R-LLD was measured from the preoperative anteroposterior radiograph as aforementioned. The Crowe classification was evaluated according to a previous report [14]. The global femoral offset (GFO) was measured by adding the distance between the longitudinal axis of the femur and the center of the femoral head to the distance between the center of the femoral head and a perpendicular line passing through the pubic symphysis [13] (Fig. 3). Computed tomography (CT) images were reviewed to evaluate the anatomical femoral anteversion (AFA) according to previously described methods [15, 16] (Fig. 4). All CT scans were acquired with the patient supine on the gantry with their feet in a comfortable resting position. Images were obtained from the superior iliac crest to the proximal tibia.

The lumbar scoliosis angle was defined as the angle between the superior border of the first lumbar vertebra and the bilateral lines passing through the upper edge of the iliac bone. A positive value was assigned to a lumbar scoliosis angle that was convex towards the side of the affected hip. The pelvic obliquity was measured as the angle between the inter-teardrop line and the horizontal line on an antero-posterior radiograph of a standing patient. A positive value was assigned to a pelvic oblique angle that was convex towards the affected hip [17]. For the radiographic evaluation of coronal spinal alignment, we measured the lumbar scoliosis angle and pelvic obliquity using radiographic images of the frontal view of the entire spine with the patient in the standing position. To assess the inter-observer reliability of R-LLD, GFO, AFA, pelvic oblique angle, and lumbar scoliosis angle, 40 hips were randomly selected and assessed by two surgeons. The inter-observer reliability values for the R-LLD, GFO, AFA, pelvic oblique angle, and lumbar scoliosis angle were 0.90, 0.84, 0.89, 0.89, and 0.90, respectively.

### Statistical analysis

A comparative analysis was performed between group E and group S. The age, BMI, JOA score, JHEQ score, P-LLD, GFO, AFA, pelvic oblique angle, lumbar scoliosis angle, and R-LLD were compared using the t-test. The chi-square test was used to analyze sex, Crowe classification, contralateral hip joint condition, history of surgery of the affected hip joint, and history of contralateral hip joint surgery. A power analysis indicated that 15 participants were required with the pelvic oblique angle as the main result. In the univariate analysis, *P* < 0.05 was defined as having a significant difference. The multivariate logistic analysis was performed by adding the age, sex, and BMI to the factors when a significant difference was found. All statistical analyses were performed using SPSS version 21 (IBM, Chicago, USA), and a *P*-value of ≤ 0.05 was considered statistically significant.

## Results

There were 28 male and 92 female patients, with an average age at surgery of 62.8 years (range, 25–85 years) and average BMI of 24.0 kg/m^2^ (range, 15.9–36.4 kg/m^2^). Group E included 92 patients (76.7%), while group S included 28 (23.3%). There were no significant differences in the age, sex, and BMI between the two groups (*P* = 0.482, 0.803, and 0.394, respectively). The number of patients with a history of hip surgery on the affected side was significantly higher in group S than in group E (32.1% vs. 10.8%, respectively; *P* = 0.015). There were no significant differences between the two groups in terms of Crowe classification, contralateral hip condition, or history of contralateral hip surgery. The P-LLD was 6.5 (-30 to 40) and 4.6 (-5 to 20) mm in groups E and S, respectively; therefore, no significant difference was observed between the two groups (Table 1).

**Table 1:** Patients demographics

Basic characteristics	Group E (*N* = 92)	Group S (*N* = 28)	*P* values
Age (years)	63.4 ± 11.7	61.6 ± 14.1	0.482
Sex (male/female)	21/71	7/21	0.803
Body mass index (kg/m^2^)	23.8 ± 3.9	24.5 ± 3.9	0.394
Crowe type I/II, n	80/12	22/6	0.363
Contralateral hip; healthy/OA/THA, n	42/34/16	17/8/3	0.433
Prior hip surgery, n (%)	10 (10.8)	9 (32.1)	0.015*
Prior hip surgery (contralateral)	7 (7.6)	4 (14.3)	0.28
Perceived leg length discrepancy (mm)	6.5 ± 8.8	4.6 ± 7.1	0.305

^*^ *P* < 0.05

Values are shown as the mean (SD) for age, body mass index, Perceived leg length discrepancy

The JOA pain scores were significantly higher in group S than in group E (21.7 ± 7.0 vs. 17.5 ± 8.2; respectively; *P* = 0.036). No significant difference was found between the two groups in terms of ROM, gait, ADL, total scores, and all domains of the JHEQ (Table 2).

**Table 2:** Clinical evaluations

	Group E (*N* = 92)	Group S (*N* = 28)	*P* values
JOA score			
Pain	17.5 ± 8.2	21.7 ± 7.0	0.036*
ROM	13.2 ± 4.7	12.0 ± 3.7	0.310
Gait	11.6 ± 3.7	11.6 ± 4.4	0.988
ADL	11.9 ± 3.4	12.4 ± 3.5	0.586
Total	54.3 ± 14.5	57.7 ± 13.1	0.333
JHEQ			
Pain	8.6 ± 6.0	11.0 ± 6.7	0.165
Movement	5.5 ± 5.3	4.8 ± 4.7	0.648
Mental	9.4 ± 6.5	10.8 ± 2.6	0.399
Total	23.5 ± 14.4	26.6 ± 11.1	0.419
Satisfaction score	79.5 ± 24.8	86.3 ± 11.4	0.306

^*^ *P* < 0.05, ** *P* < 0.01 significant difference; *JOA* Japanese Orthopaedic Association, *ROM* range of motion, *ADL* activities of daily living, *JHEQ* Japanese Orthopaedic Association Hip-Disease Evaluation Questionnaire, *VAS* Values are shown as the mean (SD)

Comparing the radiographic evaluations, the pelvic oblique angle and R-LLD were significantly larger in group S than in group E (2.9 ± 2.5° vs. 0.3 ± 2.3°, respectively; and 17.2 ± 8.9 vs. 6.3 ± 8.4 mm, respectively; both *P* < 0.01). No significant differences were found between the two groups in terms of GFO, AFA, and lumbar scoliosis angle (Table 3).

**Table 3:** Radiographic evaluations

	Group E (*N* = 92)	Group S (*N* = 28)	*P* values
Global femoral offset (mm)	143.5 ± 11.0	142.8 ± 11.4	0.78
Anatomical femoral anteversion (°)	20.1 ± 16.6	18.0 ± 17.5	0.573
Pelvic oblique angle (°)	0.3 ± 2.3	2.9 ± 2.5	< 0.01**
Lumbar scoliosis angle (°)	6.3 ± 8.5	5.9 ± 6.6	0.804
Radiographic leg length discrepancy (mm)	6.3 ± 8.4	17.2 ± 8.9	< 0.01**

^*^ *P* < 0.05, ** *P* < 0.01 significant difference

Values are shown as the mean (SD)

The multivariate logistic analysis was performed by adding age, sex, and BMI to the factors which showed a significant difference (the history of hip surgery on the affected side, JOA pain score, pelvic oblique angle, and R-LLD). On analysis, the pelvic oblique angle (odds ratio [OR]: 1.80, 95% confidence interval [CI]: 1.28–2.52; *P* < 0.01) and R-LLD (OR: 2.75, 95% CI: 1.24–6.12; *P* = 0.013) were found to be independent risk factors of inconsistent LLD (Table 4).

**Table 4:** Results of multivariate analyses

Parameters	Odds Ratio	95% CI	*P* values
Age	0.95	0.90–1.01	0.120
Sex	0.94	0.19–4.63	0.935
Body mass index	1.10	0.90–1.34	0.357
Prior hip surgery	2.39	0.45–12.7	0.307
JOA (Pain)	1.08	0.99–1.18	0.061
Pelvic oblique angle	1.80	1.28–2.52	< 0.01**
Radiographic leg length discrepancy	2.75	1.24–6.12	0.013*

95% CI: 95% confidence interval. * *P* < 0.05, ** *P* < 0.01 significant difference between means for the natural perception group and the artificial perception group. *JOA* Japanese Orthopaedic Association

We performed power analysis to determine the ability of our study to demonstrate the true difference in the pelvic oblique angle between group S and group E. The standard deviation was 2.5. We set α = 0.05, β = 0.8. To achieve this, 15 patients were required in each group.

## Discussion

In this study, we investigated the inconsistency between the R-LLD and P-LLD in patients with DHO and assessed the associated risk factors. In our study, the frequency of inconsistency between the R-LLD and P-LLD was 23.3%, and the independent risk factors associated with such inconsistency were the pelvic oblique angle and a long R-LLD.

Regarding the inconsistency between the R-LLD and P-LLD, Wylde et al. reported that P-LLD was found in 36% of cases in which the R-LLD was not found after THA [8]. In addition, Lazzanec et al. reported that the inconsistency between the R-LLD and P-LLD after THA is 60%. Some patients complain of a P-LLD, even when the postoperative R-LLD is uniform [18]. This could perhaps occur because of the inconsistency between the P-LLD before surgery and R-LLD. In addition, Fujita et al. reported the need to investigate the preoperative factors that cause a P-LLD [9]. However, there are no reports that evaluate the relationship between the R-LLD and P-LLD in patients with DHO.

In this study, the pelvic oblique angle was one of the factors that caused the inconsistency in LLD. Regarding the relationship between the pelvic oblique angle and LLD, Fujita et al. reported that patients in whom the postoperative limb was perceived to be longer had a significantly larger pelvic oblique angle than those in whom a P-LLD was not perceived [9]. Moreover, Koga et al. reported that the P-LLD after surgery was significantly greater in cases where the pelvis was tilted towards the affected side before surgery [7]. This study also suggests that the pelvic oblique angle may be related to the P-LLD, which may be attributed to the correction of LLD by tilting the pelvis to the side with a shorter leg length. When considering the correction of LLD, it is desirable to also examine the X-ray of the spine.

Another risk factor for inconsistent LLD is a long R-LLD. Adams et al. reported that patients who experienced a P-LLD after surgery had a significantly longer R-LLD initially than those who did not [19]; a long R-LLD is common in patients with DHOs. It may be corrected by pelvic tilt and tension of the soft tissue around the hip joint, resulting in inconsistency with the P-LLD. In cases with long R-LLD, R-LLD and P-LLD may be unequal, so it is necessary to consider not only the R-LLD but also the P-LLD when correcting LLDs.

In this study, the JOA pain score was significantly higher in group S in relation to the inconsistent LLD and QOL. Although no significant differences were observed in ROM, gait, ADL, total scores, and all domains of the JHEQ, the QOL score was higher in group S than in group E. We believe that this was the result of improved load environment due to the long R-LLD from tilting the pelvis. The long-term residual LLD affects the lumbar spine, knee joint, and ankle joint due to the abnormal load environment [4]. Over the long term, the patients with LLD can develop knee and ankle joint disorders on the healthy side and experience significantly deteriorating QOL. It has been reported that the use of a shoe lift may effectively treat the LLD in DHO [20, 21]; however, there are no reports of whether the R-LLD or P-LLD should be considered when opting for this treatment method. From this result, if the LLD is adjusted by shoe lift according to the R-LLD, the affected limb is excessively corrected as in Group S, and the patient may feel that the adjusted foot is longer. It may not be a satisfactory conservative treatment. It is preferable to adjust the LLD by shoe lift according to the P-LLD.

This study has several limitations. First, the number of cases is small. In particular, eight patients who felt that the P-LLD was longer than the R-LLD were excluded from this study and thus could not be examined. However, the analysis of the data from this group could be significant. In future studies, we would like to increase the number of cases included in this group. Second, we considered the R-LLD only on the frontal hip radiographs. It has been reported that the lengths of the left and right femurs differ based on the degree of subluxation in patients with DHO, and it may be preferable to evaluate the total length of the lower limbs [22]. Third, we have not directly examined the effectiveness of shoe lifts for LLD. Prospective research is needed to confirm the effects on QOL, which may be explored in future studies. Finally, we did not perform postoperative evaluations. Prospective research is needed in the future to assess the effect of a large pelvic oblique angle and long R-LLD, which caused the discrepancy between R-LLD and P-LLD in this study, on LLD after THA.

## Conclusions

We investigated the relationship between the R-LLD and P-LLD in patients with DHO. The inconsistency ratio between the R-LLD and P-LLD was 23.3% in DHO cases. A large pelvic oblique angle and long R-LLD were risk factors of inconsistent R-LLD-and-P-LLD.

Overall, hip surgeons should consider P-LLD rather than R-LLD to understand the need for conservative intervention.

## Data Availability

The datasets generated and/or analyzed during the current study are not publicly available due to their containing information that could compromise the privacy of research participants but are available from the corresponding author on reasonable request.

## Abbreviations

LLDs: Leg length discrepancy
R-LLD: Radiographic leg length discrepancy
P-LLD: Perceived leg length discrepancy
DHO: Dysplastic hip osteoarthritis
QOL: Quality of life
THA: Total hip arthroplasty

## References

[1] Richmond J, Hunter D, Irrgang J, Jones MH, Snyder-Mackler L, Van Durme D (2010). American Academy of Orthopaedic Surgeons clinical practice guideline on the treatment of osteoarthritis (OA) of the knee. J Bone Joint Surg Am.

[2] Desai AS, Dramis A, Board TN (2013). Leg length discrepancy after total hip arthroplasty: a review of literature. Curr Rev Musculoskelet Med.

[3] Konyves A, Bannister GC (2005). The importance of leg length discrepancy after total hip arthroplasty. J Bone Joint Surg Br.

[4] Murray KJ, Azari MF (2015). Leg length discrepancy and osteoarthritis in the knee, hip and lumbar spine. J Can Chiropr Assoc.

[5] Giles LG, Taylor JR (1984). The effect of postural scoliosis on lumbar apophyseal joints. Scand J Rheumatol.

[6] Golightly YM, Allen KD, Renner JB, Helmick CG, Salazar A, Jordan JM (2007). Relationship of limb length inequality with radiographic knee and hip osteoarthritis. Osteoarthritis Cartilage.

[7] Koga D, Jinno T, Okawa A, Morita S, Shinomiya K (2009). The effect of preoperative lateral flexibility of the lumbar spine on perceived leg length discrepancy after total hip arthroplasty. J Med Dent Sci.

[8] Wylde V, Whitehouse SL, Taylor AH, Pattison GT, Bannister GC, Blom AW (2009). Prevalence and functional impact of patient-perceived leg length discrepancy after hip replacement. Int Orthop.

[9] Fujita K, Kabata T, Kajino Y, Tsuchiya H (2020). Optimizing leg length correction in total hip arthroplasty. Int Orthop.

[10] Woolson ST, Harris WH (1985). A method of intraoperative limb length measurement in total hip arthroplasty. Clin Orthop Relat Res.

[11] Kuribayashi M, Takahashi KA, Fujioka M, Ueshima K, Inoue S, Kubo T (2010). Reliability and validity of the Japanese orthopaedic association hip score. J Orthop Sci.

[12] Seki T, Hasegawa Y, Ikeuchi K, Ishiguro N, Hiejima Y (2013). Reliability and validity of the Japanese Orthopaedic Association Hip Disease Evaluation Questionnaire (JHEQ) for patients with hip disease. J Orthop Sci.

[13] Crowe JF, Mani VJ, Ranawat CS (1979). Total hip replacement in congenital dislocation and dysplasia of the hip. J Bone Joint Surg Am.

[14] Kabata T, Kajino Y, Inoue D, Ohmori T, Yoshitani J, Ueno T (2019). Safety range for acute limb lengthening in primary total hip arthroplasty. Int Orthop.

[15] Nakahara I, Takao M, Sakai T, Nishii T, Yoshikawa H, Sugano N (2011). Gender differences in 3D morphology and bony impingement of human hips. J Orthop Res.

[16] Uemura K, Takao M, Otake Y, Koyama K, Yokota F, Hamada H (2018). Can anatomic measurements of stem anteversion angle be considered as the functional anteversion angle?. J Arthroplasty.

[17] Miyagi M, Fukushima K, Inoue G, Nakazawa T, Imura T, Saito W (2019). Hip-spine syndrome: cross-sectional-study of spinal alignment in patients with coxalgia. Hip Int.

[18] Lazennec JY, Brusson A, Rousseau MA, Robbins CB, Pour AE (2016). Do patients' perceptions of leg length correlate With standing 2- and 3-dimensional radiographic imaging?. J Arthroplasty.

[19] Adams CT, O'Leary RE, Gheewala RA, Roberts JT (2021). Evolving patient perception of limb length discrepancy following total hip arthroplasty. J Arthroplasty.

[20] Reid DC, Smith B (1984). Leg length inequality: a review of etiology and management. Physiother Can.

[21] Moseley CF (1996). Leg length discrepancy and angular deformity of the lower limbs. Lovell and Winter’s pediatric orthopaedics.

[22] Gallo MC, Chung BC, Tucker DW, Piple AS, Christ AB, Lieberman JR (2021). Limb Length Discrepancy in Total Hip Arthroplasty: Is the Lesser Trochanter a Reliable Measure of Leg Length?. J Arthroplasty.

